# Inhibition of HMGB1/NF-κB signaling restores Th17/Treg balance via dendritic cell modulation in liver transplant rejection

**DOI:** 10.3389/fimmu.2025.1649366

**Published:** 2025-09-04

**Authors:** Linyu Li, Jie Wang, Liyun Huang, Yi Chen, Lihong Chen

**Affiliations:** ^1^ Department of Pathology and Institute of Oncology, The School of Basic Medical Sciences, Fujian Medical University, Fuzhou, Fujian, China; ^2^ Department of Pathology, Mengchao Hepatobiliary Hospital of Fujian Medical University, Fuzhou, Fujian, China; ^3^ Diagnostic Pathology Center, Fujian Medical University, Fuzhou, Fujian, China

**Keywords:** HMGB1, dendritic cells, Th17/Treg, liver transplantation, NF-κB

## Abstract

**Background:**

Acute rejection (AR) remains a major challenge in liver transplantation (LT) despite advances in immunosuppression. High-mobility group box 1 (HMGB1) has emerged as a critical driver of immune activation; however, its role in dendritic cell (DC)-mediated T helper 17 (Th17)/regulatory T cell (Treg) imbalance during AR is unclear.

**Methods:**

Orthotopic LT was performed in rats assigned to sham, isograft, and allograft groups. Liver injury, HMGB1 expression, and hepatic DC infiltration were assessed by histopathology, immunohistochemistry, and CD11c immunofluorescence staining (IF), respectively, while serum levels of alanine aminotransferase (ALT), aspartate aminotransferase (AST), and total bilirubin (TBIL) were measured to evaluate graft function. Th17/Treg populations were analyzed by flow cytometry to assess immune imbalance. RNA sequencing (RNA-seq) was conducted to explore transcriptional changes in bone marrow-derived DCs stimulated with HMGB1 or PBS. DC maturation, cytokine secretion (ELISA), antigen uptake, and metabolic activity (CCK-8 assay) were assessed. A DC-CD4^+^ T cell coculture system was used to evaluate the ability of DCs to drive T cell proliferation and polarization. NF-κB signaling activation was examined by western blot (WB) and IF, and the NF-κB inhibitor helenalin was used to assess pathway relevance.

**Results:**

Allograft recipients displayed elevated serum ALT/AST/TBIL, accompanied by aggravated liver injury, increased rejection activity index (RAI) scores, and upregulated HMGB1 expression. While CD11c IF demonstrated a pronounced increase in hepatic DC infiltration. Th17 cell frequencies and the Th17/Treg ratio were markedly increased, while Treg proportions were reduced. RNA-seq of DCs revealed HMGB1-induced transcriptional reprogramming with nominal enrichment of NF-κB signaling, which was further confirmed by WB and IF. HMGB1 stimulation promoted DC maturation, enhanced pro-inflammatory cytokine production, and impaired antigen uptake and metabolic function. These activated DCs further facilitated CD4^+^ T cell proliferation and skewed differentiation toward the Th17 lineage while suppressing Treg induction. Notably, helenalin treatment effectively attenuated DC activation, restored their antigen uptake and metabolic activity, and reversed the Th17/Treg imbalance mediated by HMGB1-activated DCs.

**Conclusion:**

HMGB1 drives DC-mediated Th17/Treg imbalance during LT rejection through NF-κB activation. Targeting this pathway may offer a novel immunomodulatory strategy for managing AR.

## 1 Introduction

Liver transplantation (LT) remains the definitive treatment for end-stage liver disease. However, T cell-mediated acute cellular rejection (TCMR) continues to pose a major clinical challenge, affecting approximately 10–30% of recipients and significantly compromising long-term graft survival (1, 2). According to 2023 data from the United States, 30.3% of adult liver transplant recipients will require additional immunosuppressive therapy (2). Yet, long-term immunosuppression is associated with increased risk of *de novo* malignancies, particularly virus-related cancers such as post-transplant lymphoproliferative disorder (PTLD), skin cancer, and gastrointestinal tumors (2–4). These limitations underscore the urgent need for novel immunomodulatory strategies that can effectively regulate immune responses while minimizing adverse effects (1).

High-mobility group box 1 (HMGB1) is a ubiquitous non-histone chromatin-binding protein that primarily resides in the nucleus, where it regulates DNA replication, repair, and transcription (5–7). Under pathological conditions, HMGB1 can be passively released from necrotic cells or actively secreted by immune cells, acting as a damage-associated molecule pattern (DAMP) that triggers and amplifies inflammatory responses (6, 8, 9). Research has shown that HMGB1 interacts with Toll-like receptor 4 (TLR4) and receptor for advanced glycation end-products (RAGE), leading to the subsequent secretion of interleukin-6 (IL-6) and tumor necrosis factor-alpha (TNF-α) (10–12). This inflammatory cascade promotes T helper 17 (Th17) differentiation while inhibiting regulatory T cell (Treg) development, thereby exacerbating immune dysregulation and contributing to transplant rejection (13–15).

Dendritic cells (DCs) are highly specialized antigen-presenting cells (APCs) that play a pivotal role in linking innate and adaptive immunity by efficiently capturing, processing, and presenting antigens to activate CD4^+^ and CD8^+^ T cells (16, 17). Previous studies have suggested that HMGB1 significantly affects DC maturation through various signaling pathways, notably the nuclear factor-κB (NF-κB) pathway (18, 19). NF-κB is central to inflammation and immune responses, enhancing DC maturation and promoting pro-inflammatory cytokine secretion, thereby exacerbating transplant rejection (20–24).

Although the involvement of Th17/Treg imbalance in transplant rejection has been previously reported, the upstream regulatory mechanisms have not been fully elucidated. In particular, the mechanistic role of HMGB1-induced NF-κB activation in DCs, and its direct impact on T cell polarization within the transplant context, has not been systematically investigated. In this study, we established an orthotopic rat LT model to systematically investigate the role of HMGB1-mediated NF-κB activation in DCs during acute rejection (AR). We observed that increased HMGB1 expression was associated with AR and disruption of T cell homeostasis. Through comprehensive *in vitro* assays, we further demonstrated that HMGB1 activates the NF-κB pathway, promoting DC maturation and Th17 polarization while suppressing Treg differentiation. Importantly, pharmacological inhibition of NF-κB effectively reversed these effects, restoring the Th17/Treg balance. This study systematically delineates a complete immunological cascade from HMGB1-driven NF-κB activation in DCs to the subsequent imbalance in Th17/Treg populations, highlighting NF-κB as a critical, reversible target. Our findings provide novel mechanistic insights into immune dysregulation during AR and suggest that targeting the HMGB1–NF-κB axis may serve as a promising immunoregulatory approach to mitigate rejection while reducing dependence on broad-spectrum immunosuppression.

## 2 Material and methods

### 2.1 Experimental animals

Healthy male Lewis and Brown Norway (BN) rats (6–8 weeks old, 180–220 g) were obtained from the Animal Experiment Center of Fujian Medical University (Fuzhou, China). Animals were housed under specific pathogen-free (SPF) conditions (temperature, 22–25°C; humidity, 50–60%, 12-hour light/dark cycle) with free access to food and water. All experimental protocols were approved by the Institutional Animal Care and Use Committee (IACUC) of Fujian Medical University (Approval No. IACUC FJMU 2024-Y-0540) and performed in accordance with the National Institutes of Health Guide for the Care and Use of Laboratory Animals.

### 2.2 Liver transplantation model and surgical procedure

An orthotopic LT model was established using a modified two-cuff technique, as previously described (25). Animals were randomly divided into three groups (n = 6 per group) (I): sham group, which underwent laparotomy without graft transplantation (II); isograft group (iso-group), BN rats received syngeneic grafts from BN donors; and (III) allograft group (allo-group), which received grafts from Lewis donors to induce acute rejection.

### 2.3 Histological, immunohistochemical and detection of Th17 and Treg cells

On postoperative day 7, the liver grafts were harvested for histological and immunohistochemical analyses, including hematoxylin and eosin (H&E) staining and immunohistochemistry (IHC), as previously described (26, 27). Tissue sections were incubated with a rabbit anti-HMGB1 antibody (1:200; Proteintech, China). AR severity was assessed in a blinded manner using the Banff Rejection Activity Index (RAI, scores 0–9) based on portal inflammation, bile duct injury, and venous endothelial inflammation. Images were captured using a microscope (Nikon, Japan), and HMGB1 expression was quantified by the average optical density (AOD) using the ImageJ software (NIH, USA).

Recipient splenic lymphocytes were isolated using Ficoll-Paque density gradient centrifugation (density: 1.077 g/mL; Solarbio, China), and subsequently stimulated for 12 hours at 37 °C with Cell Stimulation Cocktail (Thermo Fisher Scientific, USA) diluted 1:500. The final concentrations were: phorbol 12-myristate 13-acetate (PMA, 81 nM), ionomycin (1.34 μM), brefeldin A (10.6 nM), and monensin (2 nM). After stimulation, cells were washed with PBS and stained with surface antibodies—anti-CD4-FITC and anti-CD25-PE (1:200 dilution each; Invitrogen, USA)—at room temperature for 30 min in the dark. Cells were fixed and permeabilized using Fixation/Permeabilization Buffer (Invitrogen, USA), followed by intracellular staining with anti-IL-17A-PE-Cy7 and anti-Foxp3-APC antibodies (both 1:200; Invitrogen, USA) at room temperature for 40 min. The stained cells were analyzed using flow cytometer (FCM, BD Biosciences, USA). Compensation controls were used for accurate gating and compensation adjustment.

Data were analyzed using FlowJo software (version 10.8.1, BD Biosciences, USA). The percentages of Th17 (CD4^+^IL-17A^+^) and Treg (CD4^+^CD25^+^Foxp3^+^) cells were calculated based on CD4^+^ T cells, and the Th17/Treg ratio was derived accordingly. All experiments were conducted in triplicate.

### 2.4 Isolation, culture, and characterization of BMDCs

Bone marrow cells were collected from the femurs and tibias of Lewis rats under aseptic conditions. Red blood cells were removed using red blood cell (RBC) lysis buffer (Solarbio, China). Cells were then cultured in RPMI-1640 medium (Gibco, USA) supplemented with 10% fetal bovine serum (FBS, WISENT, China), penicillin–streptomycin (P/S, 100 U/mL and 0.1 mg/mL, respectively; Biosharp, China), 20 ng/mL GM-CSF, and 10 ng/mL IL-4 (PeproTech, USA for both). Cultures were maintained at 37 °C with 5% CO_2_, and half of the medium was refreshed every other day. Immature bone marrow-derived dendritic cells (BMDCs) were harvested on days 6–7 for downstream assays.

On day 7, BMDCs were treated for 48 hours under the following conditions: PBS (5 μL/mL; Servicebio, China) control, lipopolysaccharide (LPS, 500 ng/mL; Beyotime, China), recombinant HMGB1 (3 μg/mL; Abcam, USA), or pretreatment for 1 hour with helenalin (10 μM; KKL, USA) prior to HMGB1 stimulation. For phenotypic analysis, BMDCs were stained with anti-MHC-II-APC (1:400), anti-CD80-PE (1:200), and anti-CD86-FITC (1:200) antibodies (all from Invitrogen, USA) at 4°C for 40 min and analyzed by FCM.

### 2.5 Antigen uptake assay

For the antigen uptake assays, BMDCs (1 × 10^6^ cells/mL) were incubated with FITC-labeled ovalbumin (FITC-OVA, 5 μg/mL; Solarbio, China) at 37°C (active uptake) or 4°C (negative control) for 4 hours. After incubation, the cells were washed and stained with anti-MHC-II-APC antibody (1:400) for 40 min at 4°C in the dark. Samples were analyzed using FCM and antigen uptake capacity was quantified as the difference in median fluorescence intensity (ΔMFI = MFI at 37°C – MFI at 4°C).

### 2.6 CCK-8 cell viability assay

Cells were seeded in 96-well U-bottom plates (Corning, USA) incubated at 37°C with 5% CO_2_ for 24 hours. Various concentrations of test compounds were then added, with blank (medium only) and control (untreated cells) wells included. After 48 hours of treatment, 10 μL of CCK-8 solution (YEASEN, China) was added to each well and incubated for 1 hour. Absorbance at 450 nm was measured using a microplate reader, and cell viability was calculated as: (OD sample – OD blank)/(OD control – OD blank) × 100%.

### 2.7 CD4^+^ T cells purification and co-cultured with BMDCs

Spleens from BN rats were processed into single-cell suspensions, and mononuclear cells were isolated using lymphocyte separation medium via density gradient centrifugation. CD4^+^ T cells were positively selected using CD4 MicroBeads and a MACS separation system (Miltenyi Biotec, Germany) according to the manufacturer’s instructions. The purity of isolated CD4^+^ T cells was assessed by FCM, and samples with >95% purity were used for subsequent experiments (**Supplementary Figure 1**).

BMDCs from each group (1 × 10^4^ cells/well) were seeded into 96-well U-bottom plates, and co-cultured with purified CD4^+^ T cells (1 × 10^5^ cells/well) at a 1:10 DC:T ratio. Cells were maintained in RPMI-1640 medium supplemented with 10% FBS, 1% P/S, and recombinant IL-2 (10 ng/mL, yeasen, China). Concanavalin A (Con A, 5 μg/mL; Solarbio, China) was added to activate T cells. To maintain T cell viability, 50 μL of fresh medium containing Con A was added every 48 hours. Co-culture was maintained for 5 days. CD4^+^ T cells cultured alone under identical conditions served as the negative control group.

### 2.8 T cell clonal expansion and proliferation assay

Purified CD4^+^ T cells were labeled with 4 μM carboxyfluorescein succinimidyl ester (CFSE; Invitrogen, USA) at 37°C for 5 minutes in the dark. The labeling reaction was quenched by adding an equal volume of RPMI-1640 medium containing 5% bovine serum albumin (BSA, Solarbio, China) and incubating for an additional 5–6 minutes at room temperature. Cells were then washed twice with RPMI-1640 medium to remove unbound CFSE and subsequently co-cultured with BMDCs from each group for 5 days.

CFSE dilution was measured by FCM to assess CD4^+^ T cell proliferation. The proliferation index (PI) was calculated using FlowJo software.

### 2.9 Treg and Th17 cell differentiation

For differentiation analyses of Th17 and Treg cells, CD4^+^ T cells (unlabeled) were co-cultured with DCs under the conditions described above in 96-well plates for 5 days. The medium was refreshed every other day, and CD4^+^ T cells cultured alone served as the control. On day 5, Th17 and Treg differentiation, along with the Th17/Treg ratio, was analyzed by FCM, as described above.

### 2.10 Cytokine quantification by ELISA

Culture supernatants from BMDCs of each group were collected after stimulation, and the concentrations of IL-6 and IL-12 were quantified using ELISA kits (MEIMIAN, China), according to the manufacturer’s instructions.

### 2.11 Immunofluorescence staining

#### 2.11.1 Liver immunofluorescence staining in the rat liver transplantation

Paraffin-embedded liver sections (3–5 μm) from transplanted rats were deparaffinized with xylene, rehydrated through graded ethanol, and subjected to antigen retrieval using Tris-EDTA buffer (pH 9.0) in a pressure cooker. After cooling to room temperature, sections were washed with PBS and blocked with 3% BSA for 1 hour. Primary antibody against CD11c (1:150; Proteintech, China) was incubated overnight at 4°C, followed by incubation with Alexa Fluor 488-conjugated anti-rabbit IgG (1:200; Proteintech, China) for 1 hour at room temperature in the dark. Nuclei were counterstained with DAPI (10 μg/mL; Solarbio, China) for 10 minutes at room temperature prior to mounting.

#### 2.11.2 BMDCs immunofluorescence staining *in vitro*

BMDCs were stimulated with HMGB1 (3 μg/mL; Abcam, USA) or PBS (negative control) for 48 hours and subjected to IF as described in a previous study (18). NF-κB p65 (1:200, Invitrogen, USA) was used for staining and was incubated with Alexa Fluor 488-conjugated anti-rabbit IgG (1:300; Proteintech, China). Nuclei were counterstained with DAPI, as described above.

### 2.12 Western blot analysis

Total protein was extracted from the BMDCs using RIPA lysis buffer (Beyotime, China) supplemented with protease and phosphatase inhibitors (Roche, Basel, Switzerland). Protein concentration was determined using a BCA assay kit (Invitrogen, USA). WB analysis was conducted as previously described (27). Protein samples were separated on 10% SDS–PAGE gels (Epizyme, China) alongside a prestained protein marker (Biodragon, China), and then transferred to PVDF membranes (Millipore, USA). The membranes were incubated with primary antibodies against NF-κB p65 (1:1000, Invitrogen, USA) and β-actin (1:20000; Proteintech, China) as a loading control.

### 2.13 Biochemical examination

On postoperative day 7, blood samples were collected from recipient rats (n = 6 per group). Serum alanine aminotransferase (ALT), aspartate aminotransferase (AST) and total bilirubin (TBIL) levels were measured using a fully automated biochemical analyzer (Chemray 240, Rayto, China), following the manufacturer’s instructions.

### 2.14 RNA sequencing analysis

High-quality mRNA was enriched and sequencing libraries were constructed and sequenced on an Illumina NovaSeq 6000 platform. Differentially expressed genes (DEGs) were identified using edgeR with thresholds of | log_2_ (Fold Change) | > 1.0 and *q*-value < 0.05. To identify the key regulatory pathways associated with BMDC activation, functional enrichment analyses, including Gene Ontology (GO) and Kyoto encyclopedia of genes and genomes (KEGG) pathway assessments, were conducted. Pathways with nominal enrichment (*p* < 0.05 but *q* ≥ 0.05) were also noted based on biological relevance.

### 2.15 Statistical analysis

All statistical analyses were performed using the GraphPad Prism 9.0 (GraphPad Software, San Diego, CA, USA). Data are presented as the mean ± standard deviation (SD). Normality was assessed using the Shapiro-Wilk test before conducting parametric tests. Comparisons between two groups were performed using an unpaired Student’s t-test. For comparisons involving multiple groups, one-way analysis of variance (ANOVA) followed by Tukey’s *post hoc* test was applied. Statistical significance was set at *p*-value < 0.05. Each experiment was conducted independently at least three times to ensure reproducibility. For animal studies, a minimum of six biological replicates per group were included.

## 3 Results

### 3.1 HMGB1 disrupts Th17/Treg balance and promotes acute rejection in a rat liver transplantation model

**Figure 1:**
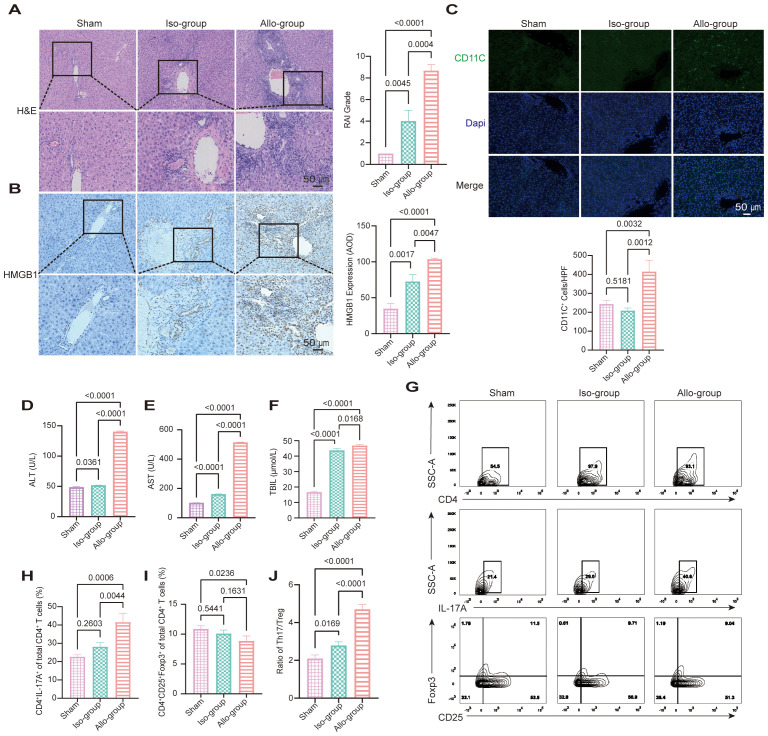
HMGB1 upregulation and Th17/Treg imbalance are associated with acute rejection in a rat liver transplantation (LT) model. **(A)** Representative hematoxylin and eosin (H&E)-stained sections of liver grafts from the sham, iso-group, and allo-group on postoperative day 7. The allo-group exhibits severe lymphocytic infiltration, bile duct injury, and endothelial inflammation. Scale bars: 50 μm. Rejection Activity Index (RAI) scores, evaluated according to Banff criteria, are significantly higher in the allo-group compared to the iso-group (*p* = 0.0004) and sham (*p* < 0.0001). **(B)** Representative immunohistochemical (IHC) staining for HMGB1 shows marked upregulation in the allo-group relative to the iso-group and sham group. Scale bars: 50 μm. Quantitative analysis of HMGB1 expression by average optical density (AOD) reveals significantly elevated levels in the allo-group versus the iso-group (*p* = 0.0047) and sham group (*p* < 0.0001). **(C)** Representative immunofluorescence (IF) images showing CD11c (green) and DAPI (blue) staining in liver sections from the sham, iso, and allo-group. Scale bar = 50 μm. Quantitative analysis of CD11c^+^ cells per high-power field (20× objective) demonstrated a significant increase in the allo-group compared with the sham group (*p* = 0.0032) and iso-group (*p* = 0.0012). **(D-F)** Serum alanine aminotransferase (ALT), aspartate aminotransferase (AST) levels, and total bilirubin (TBIL) were significantly elevated in the allo-group compared with the sham and iso-group (*p* < 0.05 for all comparisons). **(G-J)** Representative flow cytometry (FCM) plots and quantification of CD4^+^ T cells, Th17 cells (CD4^+^IL-17A^+^), and Treg cells (CD4^+^CD25^+^Foxp3^+^) from the sham, iso-group, and allo-group. The proportion of Th17 cells was significantly increased in the allo-group compared with both the iso-group (*p* = 0.0044) and sham (*p* = 0.0006). Treg frequencies were significantly reduced in the allo-group compared with the sham group (*p* = 0.0236), while no significant difference was observed between the allo-group and iso-group (*p* = 0.1631). As a result, the Th17/Treg ratio was markedly elevated in the allo-group relative to both the sham (*p* < 0.0001) and iso-group (*p* < 0.0001). Data are expressed as mean ± SD; statistical significance was determined by one-way ANOVA (*p* < 0.05).

We established an orthotopic rat LT model to investigate the role of HMGB1 in AR after LT. On postoperative day 7, liver grafts from the allograft group exhibited significant histopathological signs of AR, including increased lymphocytic infiltration, bile duct injury, and venous endothelial inflammation, as evidenced by elevated RAI scores compared with the sham and iso-group controls (*p* < 0.0001, *p* = 0.0004; **Figure 1A**). IHC analysis demonstrated marked HMGB1 upregulation in allograft tissues (**Figure 1B**). In parallel, CD11c IF revealed increased hepatic infiltration of DC in the allo-group compared with the sham and iso-group (*p* = 0.0032 and 0.0012, respectively; **Figure 1C**), indicating enhanced recruitment of antigen-presenting cells to the liver graft. These findings were consistent with severe graft injury, indicated by significantly elevated serum ALT, AST and TBIL levels (*p* < 0.05; **Figures 1D–F**).

FCM analysis revealed altered distributions of CD4^+^ T cell subsets among the experimental groups (**Figures 1G–J**). The proportion of Th17 cells (CD4^+^IL-17A^+^) was significantly increased in the allo-group compared to both the iso-group (*p* = 0.0044) and sham group (*p* = 0.0006), while the iso-group did not differ significantly from sham group (*p* = 0.2603). Treg cells (CD4^+^CD25^+^Foxp3^+^) were reduced in the allo-group compared to sham group (*p* = 0.0236), with no significant difference between allo-group and iso-group (*p* = 0.1631), or between iso-group and sham group (*p* = 0.5441). Consequently, the Th17/Treg ratio was markedly increased in the allo-group compared with both the iso-group (*p* < 0.0001) and sham group (*p* < 0.0001), and mildly elevated in the iso-group compared to sham group (*p* = 0.0169). These data indicate that allogeneic transplantation triggers a prominent Th17/Treg imbalance characterized by Th17 expansion and Treg suppression. In contrast, syngeneic transplantation maintained relatively stable CD4^+^ T cell profiles, with only a slight shift in the Th17/Treg ratio.

These results suggest that HMGB1 is a key driver of AR after LT, implicating it as a potential therapeutic target for mitigating transplant rejection by restoring immune homeostasis.

### 3.2 Transcriptomic analysis reveals HMGB1-mediated DC activation and immune modulatory potential

**Figure 2:**
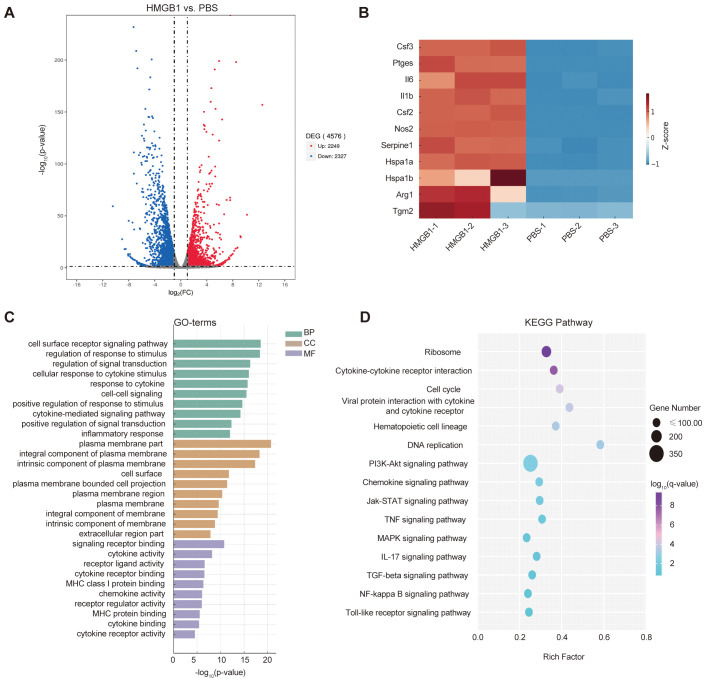
HMGB1-induced transcriptomic alterations in dendritic cells (DCs). **(A)** Volcano plot showing differentially expressed genes (DEGs) in HMGB1-treated DCs vs. PBS controls (|log_2_FC| > 1.0, *q* < 0.05), with 2,249 upregulated (red) and 2,327 downregulated (blue) genes. **(B)** Heatmap illustrating relative expression levels of selected immune-related genes involved in proinflammatory mediators (e.g., *Nos2*, *Il6, Il1b*), DC activation and cytokine signaling (e.g., *Csf2*, *Csf3*, *Ptges*), and stress response factors (e.g., *Hspa1a*, *Serpine1*). Color intensity indicates log_2_-scaled expression values. **(C)** Gene Ontology (GO) enrichment analysis identifying significantly enriched biological processes (BP), cellular components (CC), and molecular functions (MF) (*p*-value < 0.05). Enriched GO terms include immune regulation (e.g., inflammatory response, cytokine-mediated signaling pathway), plasma membrane region, extracellular region part, and molecular functions such as cytokine receptor binding and receptor-ligand activity. **(D)** KEGG pathway enrichment analysis of HMGB1-treated DCs. Immune-related pathways with strong mechanistic relevance—such as cytokine–cytokine receptor interaction, NF-κB signaling, Jak–STAT signaling, and IL-17 signaling—were highlighted. Dot size represents gene counts; color gradient indicates –log_10_(*q*-value).

To investigate how HMGB1 regulates DC function and its potential impact on T cell responses, RNA-seq was performed on DCs treated with HMGB1 and PBS controls. Differential expression analysis identified 4,576 DEGs (2,249 upregulated and 2,327 downregulated), indicating significant transcriptional alterations in HMGB1-treated DCs (**Figure 2A**). The heatmap of selected immune-related DEGs (**Figure 2B**) clearly demonstrates the elevated expression of genes associated with proinflammatory mediators (e.g., *Nos2*, *Il6, Il1b*), DC activation and cytokine signaling (e.g., *Csf2*, *Csf3*, *Ptges*), and stress response factors (e.g., *Hspa1a*, *Serpine1*). These findings collectively suggest that HMGB1 induces a robust proinflammatory transcriptional program in DCs.

GO enrichment analysis based on upregulated genes revealed significant enrichment of immune-related biological processes (BP), cellular components (CC), and molecular functions (MF, **Figure 2C**). Specifically, enriched BP included cytokine-mediated signaling pathways, inflammatory response, and positive regulation of signal transduction, indicating enhanced immunoregulatory activity upon HMGB1 stimulation. At the CC level, GO terms such as plasma membrane part, extracellular region part, and cell surface were significantly enriched, suggesting that many of the altered genes are involved in cell communication and antigen presentation. For MF, enrichment was observed in cytokine receptor binding, receptor ligand activity, and MHC class I protein binding, consistent with a heightened state of antigen processing and immune signaling in HMGB1-treated DCs.

KEGG pathway enrichment analysis based on all differentially expressed genes revealed several immune-related signaling pathways, including cytokine–cytokine receptor interaction, NF-κB signaling, Jak–STAT signaling, and IL-17 signaling (**Figure 2D**). The NF-κB signaling pathway showed nominal enrichment (*p* < 0.05) and was prioritized due to its mechanistic relevance to HMGB1-induced DC activation. As a central regulator of pro-inflammatory cytokines such as IL-6 and TNF-α, NF-κB plays a pivotal role in CD4^+^ T cell differentiation and immune homeostasis.

Overall, these transcriptomic data illustrate that HMGB1 extensively reprograms DC transcriptional networks, driving a pronounced pro-inflammatory phenotype that may disrupt T cell homeostasis. Our results define the critical transcriptional mechanisms underpinning HMGB1-induced DC activation, thereby highlighting potential therapeutic targets for modulating immune responses following LT.

### 3.3 HMGB1 activates the NF-κB pathway in DCs

**Figure 3:**
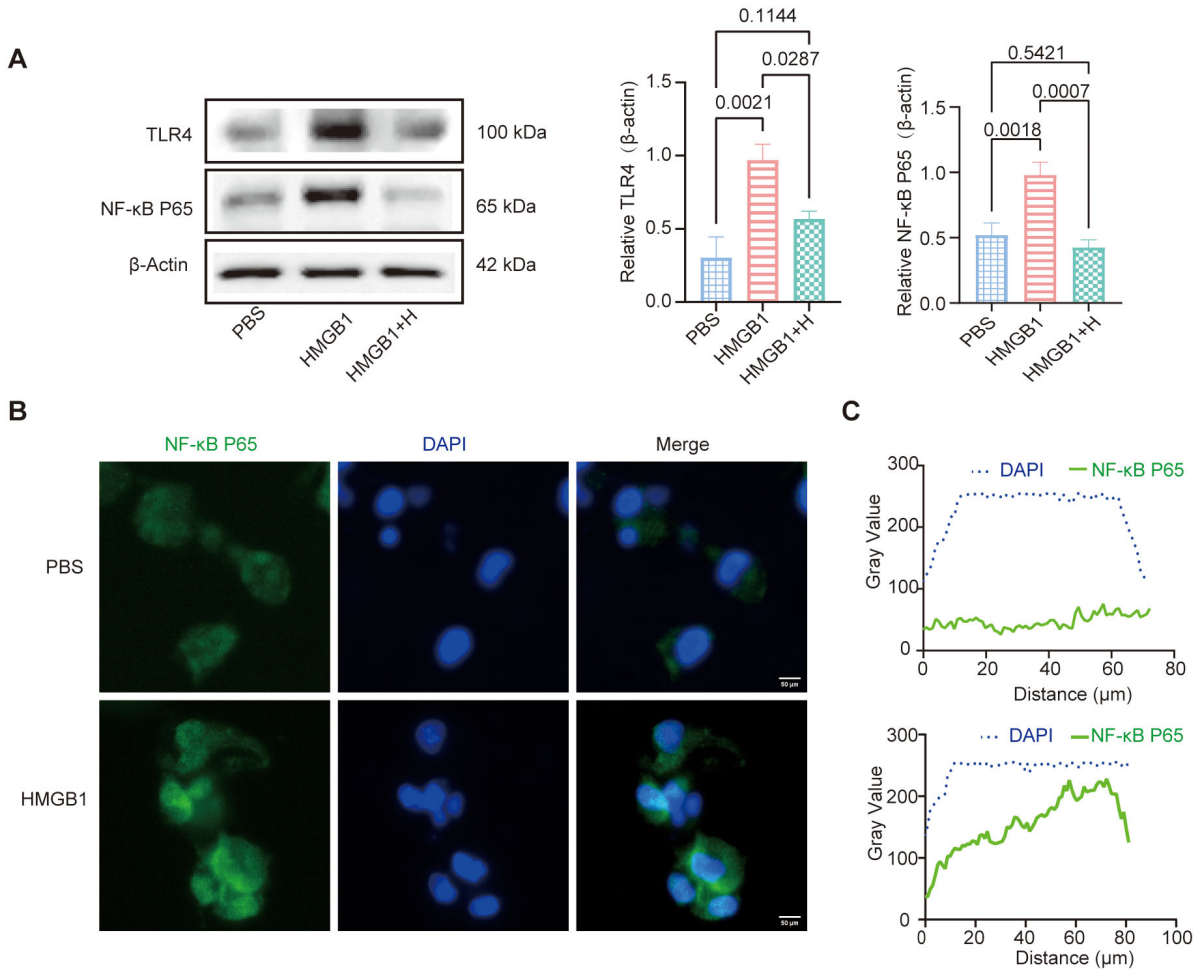
HMGB1-NF-κB axes promote DCs activation. **(A)** Western blot (WB) analysis of TLR4 and NF-κB p65 expression, with β-Actin as an internal control. Quantification of TLR4 and NF-κB p65 expression using AOD analysis. AOD revealed that HMGB1 significantly increased TLR4 (*p* = 0.0021) and NF-κB p65 (*p* = 0.0018) expression compared with PBS. Helenalin treatment markedly reduced NF-κB p65 expression (*p* = 0.0007) and moderately decreased TLR4 levels (*p* = 0.0287), though not statistically different from PBS. **(B)** IF analysis demonstrated that HMGB1 promoted NF-κB p65 nuclear translocation, as evidenced by co-localization with DAPI. Scale bars: 50 μm. **(C)** Gray value analysis confirmed an increase in nuclear NF-κB p65 signal (green). Data are presented as mean ± SD and analyzed by one-way ANOVA (*p* < 0.05).

WB analysis revealed that HMGB1 treatment significantly increased the expression of TLR4 and NF-κB p65 protein in DCs compared to PBS-treated controls (TLR4: *p* = 0.0021; NF-κB p65: *p* = 0.0018). Pretreatment with the selective NF-κB inhibitor helenalin significantly reduced the expression of both TLR4 (*p* = 0.0287) and NF-κB p65 (*p* = 0.0007), indicating that NF-κB signaling is involved in HMGB1-induced upregulation of these key inflammatory mediators (**Figure 3A**). IF staining confirmed that HMGB1 stimulation significantly enhanced the nuclear translocation of NF-κB p65, further validating the activation of the NF-κB signaling pathway (**Figures 3B, C**). Taken together, these results clearly demonstrate that HMGB1 activates the NF-κB signaling pathway in DCs and that this activation can be attenuated by NF-κB inhibition, highlighting its potential therapeutic value in modulating immune responses.

### 3.4 Inhibition of NF-κB suppresses HMGB1-mediated DC activation

**Figure 4:**
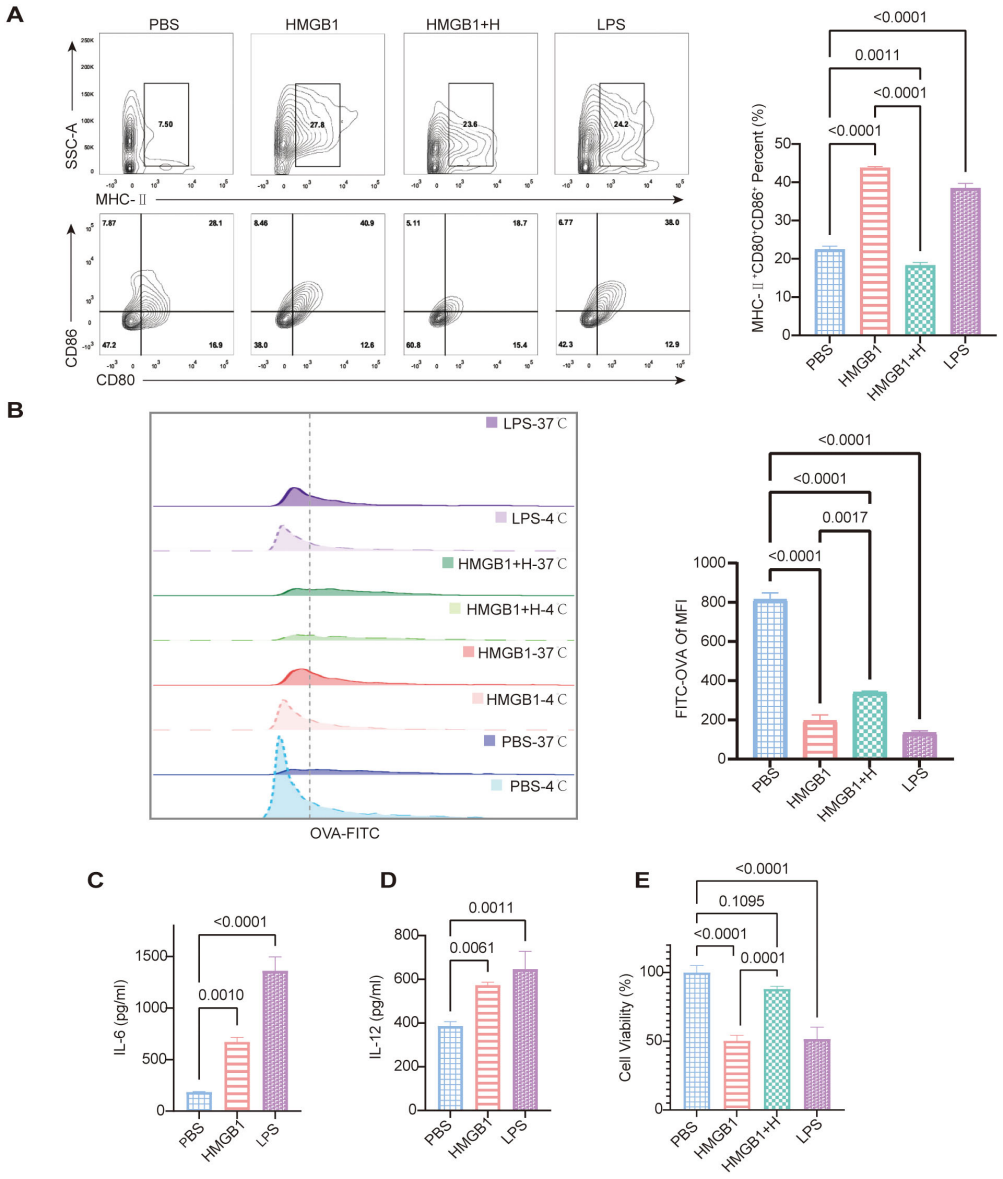
HMGB1 promotes DC maturation and impairs phagocytic function, while NF-κB inhibition reverses these effects. **(A)** FCM analysis shows increased expression of maturation markers (MHC-II, CD80, CD86) in HMGB1-treated DCs, which is reduced by helenalin. Quantification of MHC-II^+^CD80^+^CD86^+^ confirms a significant increase in the HMGB1 group vs. PBS, partially reversed by helenalin (*p* < 0.0001). LPS also significantly increases mature DCs (*p* < 0.0001). **(B)** OVA-FITC uptake assay shows reduced antigen uptake in HMGB1-treated DCs, partially restored by helenalin. FITC-OVA mean fluorescence intensity (MFI) confirms HMGB1-induced antigen uptake reduction (*p* < 0.0001), reversed by helenalin (*p* = 0.0017). **(C, D)** ELISA shows HMGB1 significantly increases IL-6 (*p* = 0.0010) and IL-12 (*p* = 0.0061); LPS-treated DCs exhibit the highest cytokine levels (*p* < 0.0001, *p* = 0.0011). **(E)** Cell viability of BMDCs was assessed using the CCK-8 assay. Both HMGB1 and LPS significantly reduced cell viability compared with PBS controls (*p* < 0.0001), while helenalin partially reversed the HMGB1-induced decrease in viability (*p* = 0.0001). Data are presented as mean ± SD and analyzed by one-way ANOVA (*p* < 0.05).

To assess the role of NF-κB signaling in HMGB1-induced DC activation, we evaluated DC morphology and expression of maturation markers. HMGB1-treated DCs exhibited increased dendritic projections and significantly upregulated expression of maturation markers, including MHC-II, CD80, and CD86, compared to controls (*p* < 0.0001). Pretreatment with the selective NF-κB inhibitor helenalin markedly suppressed these maturation changes (*p* < 0.0001). LPS, which was used as the positive control, also induced significant DC maturation (*p* < 0.0001; **Figure 4A**). Furthermore, HMGB1 stimulation significantly impaired the antigen uptake capacity of DCs, as measured by FITC-OVA uptake (*p* < 0.0001). This suppression of antigen presentation was effectively reversed by NF-κB inhibition (*p* = 0.0017; **Figure 4B**). ELISA analysis demonstrated that HMGB1 significantly elevated the secretion of pro-inflammatory cytokines IL-6 and IL-12 (both *p* < 0.05; **Figures 4C, D**).

CCK-8 assays demonstrated that DC stimulation with HMGB1 or LPS significantly reduced cell viability compared to PBS-treated controls (both *p* < 0.0001). Pretreatment with the NF-κB inhibitor helenalin effectively restored cell viability compared to HMGB1-treated DCs without inhibitor (*p* = 0.0001), indicating an NF-κB-dependent mechanism underlying these metabolic changes (**Figure 4E**). Collectively, these findings indicate that although HMGB1 and LPS do not induce substantial cell death, they markedly impair DC metabolic activity, reflecting the increased metabolic stress typically associated with cellular activation. Inhibition of NF-κB signaling by helenalin mitigated this stress, thereby partially restoring DC viability. Taken together, our results highlight a pivotal role for NF-κB in HMGB1-mediated DC maturation, pro-inflammatory cytokine secretion, antigen presentation, and metabolic function. Crucially, NF-κB inhibition effectively reversed these key immunological features of DC activation, supporting the therapeutic potential of targeting NF-κB signaling to modulate immune responses during transplantation.

### 3.5 Inhibition of NF-κB suppresses HMGB1-mediated DC activation, CD4^+^ T cell proliferation, and restores Th17/Treg balance

**Figure 5:**
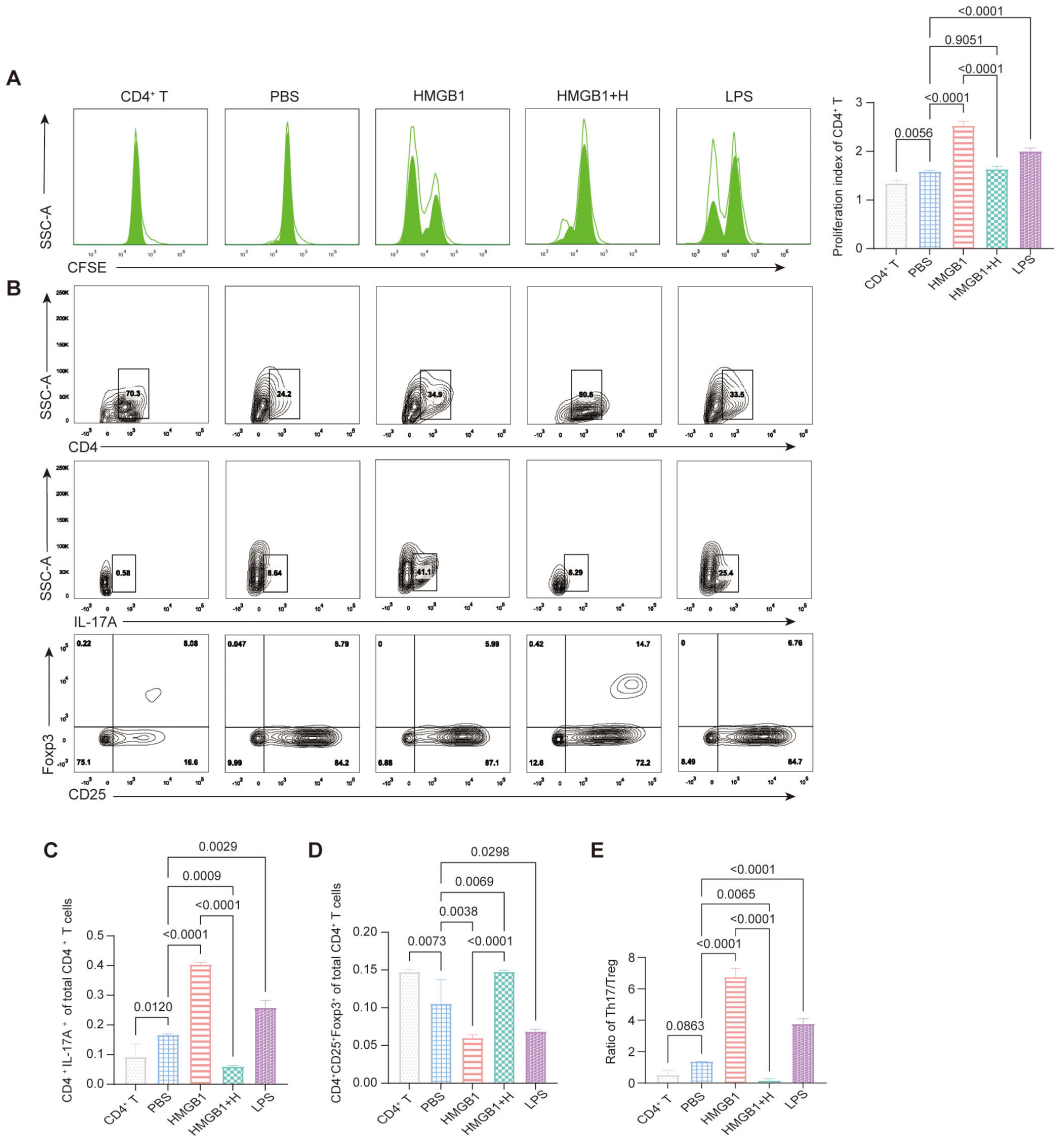
NF-κB-dependent effects of HMGB1 on DC-mediated CD4^+^ T cell proliferation and Th17/Treg differentiation. **(A)** CFSE dilution assay shows that HMGB1 enhances CD4^+^ T cell proliferation more strongly than LPS (*p* < 0.0001), while helenalin significantly suppresses HMGB1-induced proliferation (*p* < 0.0001). **(B-E)** HMGB1-treated BMDCs promote Th17 differentiation (*p* < 0.0001), with LPS inducing a moderate increase (*p* = 0.0029); helenalin inhibits HMGB1-driven Th17 polarization (*p* < 0.0001). HMGB1 reduces Treg differentiation (*p* = 0.0038), while LPS exerts a weaker inhibitory effect (*p* = 0.0298); helenalin significantly restores Treg levels (*p* < 0.0001). HMGB1 shifts the Th17/Treg ratio toward a pro-inflammatory state (*p* < 0.0001), while LPS induces a moderate imbalance (*p* < 0.0001); helenalin reverses the HMGB1-induced Th17/Treg disruption (*p* < 0.0001). Data are presented as mean ± SD and analyzed by one-way ANOVA (*p* < 0.05).

To investigate the role of NF-κB signaling in HMGB1-induced CD4^+^ T cell proliferation, we conducted co-culture assays of CD4^+^ T cells with HMGB1-stimulated DCs with or without NF-κB inhibition. Compared to PBS-treated controls, HMGB1 significantly enhanced CD4^+^ T cell proliferation (*p* < 0.0001), indicating that HMGB1 promoted DC-mediated CD4^+^ T cell activation. Notably, pre-treatment with helenalin markedly attenuated this effect (*p* < 0.0001) and restored T cell proliferation to a level comparable to that of PBS controls (*p* = 0.9051). These findings suggest that HMGB1 promotes CD4^+^ T cell proliferation via NF-κB activation in DCs, and that NF-κB inhibition effectively counteracts this immune-activating effect (**Figure 5A**).

FCM analysis (**Figure 5B**) further revealed that HMGB1-treated DCs promoted Th17 differentiation (CD4^+^IL-17A^+^; *p* < 0.0001), whereas LPS induced a smaller increase (*p* = 0.0029). NF-κB inhibition significantly attenuated HMGB1-induced Th17 polarization (*p* < 0.0001; **Figure 5C**). Conversely, Treg (CD4^+^CD25^+^Foxp3^+^) frequencies were significantly reduced in the HMGB1 group compared to the PBS controls (*p* = 0.0038), with a modest reduction observed following LPS treatment (*p* = 0.0298). NF-κB inhibition effectively restored Treg cell differentiation in HMGB1-stimulated DC coculture (*p* < 0.0001; **Figure 5D**). Consequently, HMGB1 stimulation significantly increased the Th17/Treg ratio (*p* < 0.0001), whereas LPS treatment resulted in moderate elevation (*p* < 0.0001). NF-κB inhibition reversed HMGB1-induced Th17/Treg imbalance, restoring immune homeostasis (*p* < 0.0001; **Figure 5E**). These results demonstrated that HMGB1 promotes pro-inflammatory immune responses by enhancing Th17 differentiation and suppressing Treg cell induction via NF-κB activation in DCs. NF-κB inhibition effectively counteracts these effects, underscoring its therapeutic potential for modulating immune responses during transplantation.

## 4 Discussion

HMGB1 is a well-established DAMP protein that plays a crucial role in transplant rejection and ischemia-reperfusion injury (IRI) (28), previously shown to enhance IL-6-dependent Th17 responses (29) and activate DCs in acute liver graft rejection (30). Additionally, Gal-1 regulates the DC-induced Treg/Th17 balance via NF-κB/RelB signaling (31). In the present study, we observed significant HMGB1 upregulation in liver grafts undergoing AR, correlating strongly with severe histopathological damage including lymphocytic infiltration, bile duct injury, and endothelial inflammation. IF analysis further revealed a significant increase in hepatic CD11c^+^ DC infiltration, suggesting enhanced recruitment and potential involvement of DCs in the local immune response. Taken together with prior studies identifying DCs as key targets of HMGB1-mediated immune activation (32–34), our findings uniquely demonstrate that HMGB1 disrupts Th17/Treg balance specifically via NF-κB-mediated DC activation.

Compared with other transplanted organs, the liver possesses unique tolerogenic properties and is often considered an “immune-privileged” organ (35). However, our findings suggest that liver damage, such as IRI, immune cell infiltration, or the release of necrotic hepatocyte-derived HMGB1, may compromise immune tolerance, shift the immune response toward a pro-inflammatory state, and ultimately lead to graft dysfunction. These findings underscore the pivotal role of HMGB1 in LT rejection and highlight its potential as a novel immunoregulatory target for rejection mitigation.

DCs act as a critical bridge between innate and adaptive immunity, and play a key role in transplant rejection (36, 37). Previous studies have indicated that HMGB1 activates the NF-κB signaling pathway via TLR4 and RAGE receptors (11, 38, 39); however, its detailed regulatory effects on DC-mediated immune responses remain incompletely understood. To further investigate the function of the HMGB1/NF-κB axis in DCs, we performed a transcriptomic analysis, which revealed that HMGB1-treated DCs exhibited significant enrichment of inflammatory and immune-regulatory pathways, particularly those involving NF-κB and TLR signaling. These pathways are well-established key regulators of DC activation and antigen presentation (19, 37). WB and IF analyses confirmed that HMGB1 activated the NF-κB signaling pathway by upregulating TLR4 and NF-κB p65 expression and enhancing NF-κB p65 nuclear translocation. These effects were significantly suppressed by NF-κB inhibition, directly validating the HMGB1/NF-κB axis in DC activation.

While NF-κB inhibitors have been explored in autoimmune diseases and transplant rejection, prior studies have predominantly focused on their role in T cell-or macrophage-mediated inflammatory pathways (19, 24, 40, 41), with limited investigation into their function in DC-mediated Th17/Treg balance. To assess the functional effect of NF-κB activation on HMGB1-stimulated DCs, we examined DC maturation marker expression and antigen uptake capacity. HMGB1 treatment significantly promoted DC maturation, as indicated by upregulated MHC-II, CD80, and CD86 expression, along with reduced antigen uptake capacity. These effects were effectively suppressed by helenalin, a specific NF-κB inhibitor, demonstrating the central role of NF-κB in HMGB1-induced DC maturation. Furthermore, ELISA results showed that HMGB1 increased DC secretion of IL-6 and IL-12, cytokines known to drive Th17 differentiation, and enhanced inflammatory responses (34). In parallel, CCK-8 assays revealed that treatment with HMGB1 or LPS caused a mild yet significant reduction in DC metabolic activity. This effect was partially reversed by helenalin, suggesting that the observed functional decline may result from activation-induced metabolic stress rather than direct cytotoxicity. These findings suggest that while HMGB1 enhances DC immunological functions via NF-κB signaling, it may concurrently impose a functional and metabolic burden on DCs, a feature consistent with activation-induced remodeling.

Co-culture experiments demonstrated that HMGB1-treated DCs significantly enhanced CD4^+^ T cell proliferation, promoted Th17 differentiation, and suppressed Treg induction, resulting in a skewed Th17/Treg ratio. Given the well-established role of Th17/Treg imbalance in transplant rejection and autoimmune diseases (14). Importantly, NF-κB inhibition effectively reversed HMGB1-induced Th17/Treg imbalance, restoring immune homeostasis., these findings highlight HMGB1 as a potent immunomodulator. Mechanistically, NF-κB inhibition effectively reversed the HMGB1-induced immune polarization and restored immune homeostasis, underscoring the pivotal role of the NF-κB pathway in mediating DC-driven immune dysregulation.

Currently, immunosuppressive regimens for LT primarily rely on calcineurin inhibitors such as tacrolimus, often combined with mycophenolate mofetil and corticosteroids. While effective, these broad-spectrum agents increase the risk of infection, malignancy, and metabolic complications (2). In contrast, NF-κB inhibition has been proposed as a more selective immunomodulatory strategy by modulating DC function and downstream T cell responses, potentially reducing systemic immunosuppression-related toxicity. Previous studies in heart and kidney transplantation have demonstrated that NF-κB blockade attenuates rejection and prolongs graft survival (42, 43). Our findings extend this concept to LT and provide mechanistic evidence supporting NF-κB as a candidate immunoregulatory target. However, further *in vivo* validation and pharmacological refinement are necessary to assess its clinical feasibility.

Despite these advantages, the clinical application of helenalin remains limited due to its inherent cytotoxicity. This toxicity arises from its covalent interactions with sulfhydryl groups in essential biomolecules such as glutathione, leading to broad off-target effects (44). Future strategies to overcome this limitation may include the development of less toxic helenalin analogs (45), nanoparticle-mediated targeted drug delivery specifically to DCs (46), or exploring structurally distinct NF-κB inhibitors with improved safety profiles.

One acknowledged limitation of this study is the lack of *in vivo* validation using NF-κB inhibitors, which limits direct causal confirmation between HMGB1–NF-κB signaling and Th17/Treg dysregulation during LT rejection. Although our *in vitro* experiments provide mechanistic insights and demonstrate reversibility of DC activation and T cell polarization via NF-κB inhibition, these findings alone cannot fully confirm therapeutic efficacy *in vivo*. Nevertheless, our model is indirectly supported by prior animal studies. For example, NF-κB downregulation has been shown to modulate CD4^+^ T cell polarization, particularly promoting Treg differentiation in transplant and inflammatory settings (31). Moreover, previous work has shown that inhibition of NF-κB signaling alleviates inflammation and restores Treg/Th17 balance in various immune-mediated disease models, such as sepsis-induced lung injury and asthma (47, 48). These findings provide indirect evidence that targeting NF-κB can modulate T cell polarization and ameliorate inflammatory pathology.

Future studies are warranted to investigate whether selective NF-κB inhibition improves allograft outcomes in transplantation settings and to assess its safety and therapeutic potential *in vivo*.

In conclusion, our findings provide novel mechanistic insights, establishing that HMGB1-driven NF-κB activation in DCs critically disrupts Th17/Treg immune balance during acute LT rejection. Importantly, targeted NF-κB inhibition effectively reverses this immune dysregulation, highlighting NF-κB as a promising immunotherapeutic target. Our *in vitro* experiments robustly establish a mechanistic foundation for NF-κB inhibition’s therapeutic potential; however, we acknowledge a notable limitation that our *in vivo* transplantation model did not incorporate direct NF-κB inhibitor administration.

## Data Availability

The original data presented in the study are publicly available. The RNA-seq datasets have been deposited in the Zenodo repository (DOI: 10.5281/zenodo.16886523). The original western blot images are provided in the Supplementary Material. Further inquiries can be directed to the first author (Email: lilinyu1218@163.com).
