# Supplementary material for: Inhibition of HMGB1/NF-κB signaling restores Th17/Treg balance via dendritic cell modulation in liver transplant rejection
Source: Front Immunol. 2025 Sep 4;16:1649366. doi: 10.3389/fimmu.2025.1649366 (PMC12444659; doi:10.3389/fimmu.2025.1649366)
Supplement: Supplementary file 1 [file DataSheet1.pdf]

Supplementary Figures

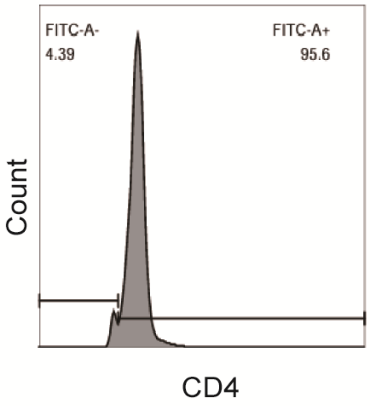

Supplementary Figure 1. The $CD4^+$ population accounted for 95.6% of the total gated cells, confirming high purity for downstream experiments. Data are representative of three independent experiments.

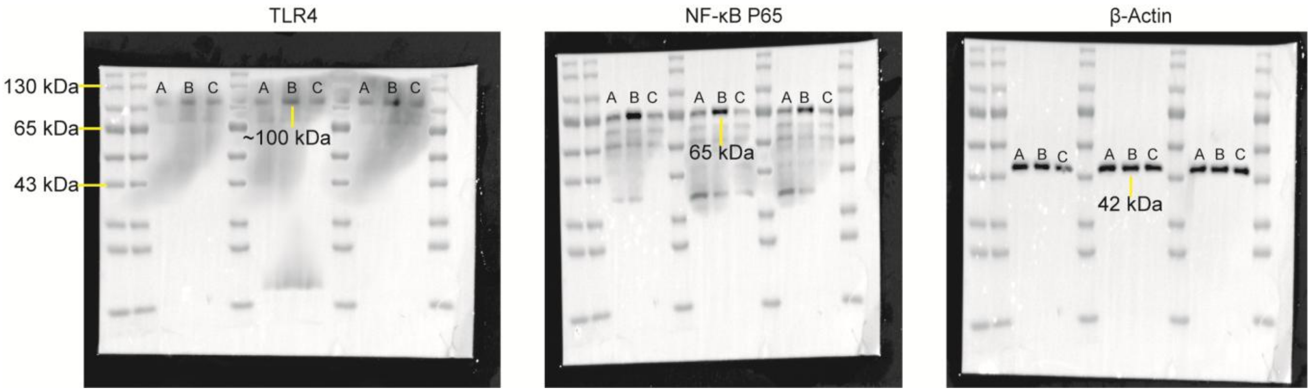

Supplementary Figure 2. Western blotting images of TLR4, NF-κB p65 and β-Actin expression in DCs treatment with PBS (A), HMGB1 (B), and HMGB1 combined with helenalin (C). Experiments were repeated three times.
